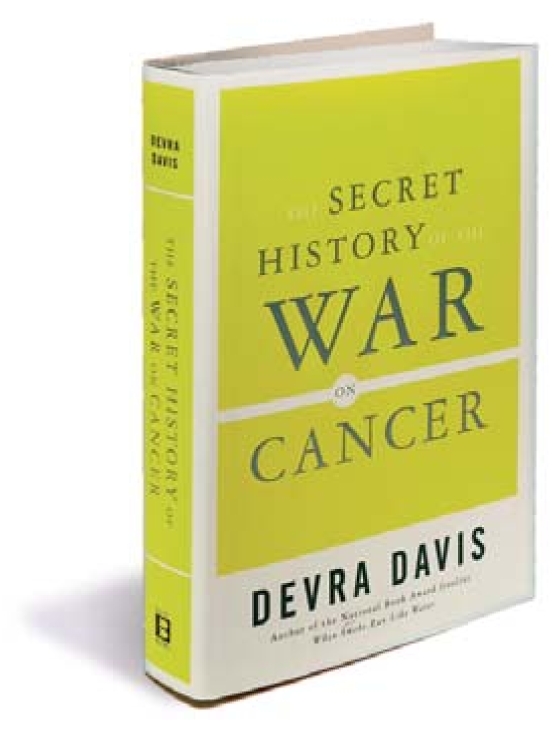# The Secret History of the War on Cancer

**Published:** 2008-02

**Authors:** James Huff

**Affiliations:** James Huff is associate director for chemical carcinogenesis, National Institute of Environmental Health Sciences

by Devra Davis

New York:Basic Books, 2007. 505 pp. ISBN: 978-0-465-01566-5, $27.95

The secret of *The Secret History of the War on Cancer* is prevention, an acutely recognized but long neglected solution to workplace-, environmental-, and public health–associated cancers. Davis begins by asking what we know and how much we need to know about suspected carcinogens before taking the actions necessary to reduce or eliminate exposures to known and suspected carcinogens. Hence prevention.

This assured strategy of preventing cancer versus treating cancer has been virtually ignored by national health and regulatory agencies, writes Davis, whereupon she describes the interconnections of industry, science, and government to maintain the status quo. Few of the hundreds of known carcinogens have been banned from use. Occupational exposure standards have been established for only a relatively small number of chemical carcinogens, and even here the chemical industry wields considerable and influential political power. Pioneering public health–minded individuals such as Lorenzo Tomatis and David Rall made great strides in primary prevention of diseases from chemicals, but their efforts were met with overwhelming resistance by government and industry to their relatively simple strategies for reducing cancer burdens. Davis presents in detail primary prevention tactics that could be easily implemented.

For example, Davis chronicles prevention efforts from the Surgeon General’s 1965 declaration that smoking causes cancer to the present, as well as feeble efforts to thwart tobacco smoking. She writes that the strength of the tobacco industry and the malfeasance of politicians and regulatory agencies combined to prevent public health action, thus condoning nearly 500,000 preventable deaths each year. She describes similar failures to prevent exposure to asbestos, lead, and industrial chemicals.

Davis details with striking historical perspective how those with interests in maintaining the use of carcinogens in industry cast doubt on epidemiologic research. Industry considers only epidemiologic evidence as potential proof of harm, even though the evidence is always vociferously challenged; conversely, industry promotes “the absence of human studies [as] proof that there was no harm.” But animal studies are rarely considered by industry or regulatory agencies as sufficient evidence to prove harm, because in their view the similarities and extrapolation to humans are not valid. As amply illustrated by Davis, these debates have intensified over time, as have the attempts to control scientific information: “What information is permitted to get to the marketplace, who decides when to release findings about public health hazards, all these things are not determined by scientific inquiry but by the social and economic realities that constrain them.” For example, in the mid-1970s when the Occupational Safety and Health Administration (OSHA) used incontrovertible human evidence of benzene-induced leukemias to reduce workplace exposures to benzene, a known carcinogen since the late 1920s, industry was able to thwart these reductions because the U.S. Supreme Court ruled that OSHA did not consider the benefits versus risks in their evaluation. Ten more years went by before the standards were strengthened.

Clearly, Davis writes, profit is the key issue. Reducing exposures reportedly costs the affected industries more money than they can afford or want to spend and still make profits. For example, workers cleaning vinyl chloride (VC) reaction vats developed the same rare form of liver cancer (hemangiosarcoma) as first seen in animals, and the plastics industry was ordered to reduce/eliminate exposures to VC—but was not required to initiate any changes until there was verifiable evidence of cancer in humans. Instead, industry stated that the risks were “small” and that the plastics industry would not survive. Losing this argument and using their ingenuity rather than further litigation, industry automated the process and eliminated the need to manually clean VC reaction vats, actually making the process more streamlined and safe as well as more cost-effective and concomitantly reducing the workers’ risks of cancer.

The real debates and struggles arise when animal data show unequivocal carcinogenicity, but with no studies available in humans. Davis notes the considerable evidence that animal cancer data do indeed predict carcinogenic risks to humans. Scientists such as Davis and regulators who believe in the value of bioassays for public health cite at least five reasons supporting the continuation of bioassays and using this information to protect the public from unnecessary exposures to carcinogens: there are more similarities among mammals (humans and rodents) than differences; all accepted human carcinogens are also carcinogenic in animals; there are common cancer sites between animals and humans; nearly one-third of the identified human carcinogens were discovered first in animal bioassays; and findings from independently conducted adequate bioassays on the same chemicals are consistent.

*The Secret History of the War On Cancer,* exhaustively researched and deftly written, illuminates more of the truth about chemicals and cancer and the relatively simple means of preventing or reducing cancer burdens. Davis emphasizes that “It’s time to admit that our efforts have often targeted the wrong enemies and used the wrong weapons.” This exposé should be required reading in toxicology courses and also be made available in high school, college, public health libraries, and libraries in general. The message is clear: To reduce cancers, one need only reduce unnecessary exposures to mutagens and carcinogens as well as to chemicals in general.

## Figures and Tables

**Figure:**